# Gallbladder Papillary Neoplasia Associated With Intrahepatic Carcinoma and Pancreaticobiliary Malformation

**DOI:** 10.4021/gr505e

**Published:** 2012-11-20

**Authors:** Vivian Resende, Rodrigo Roda, Moises Salgado Pedrosa

**Affiliations:** a Surgical Department of Minas Gerais Federal University School of Medicine, Belo Horizonte, Brazil; b Clinical Hospital of Minas Gerais Federal University School of Medicine, Belo Horizonte, Brazil; c Minas Gerais Federal University School of Medicine, Belo Horizonte, Brazil

**Keywords:** Gallbladder, Papillary neoplasia, Pancreaticobiliary maljunction

## Abstract

Papillary carcinoma is a rare tumor of the gallbladder. Papillary mucinous lesions of the intra- and extra-hepatic biliary tract (BT- IPMN) have been recognized. However the gallbladder is not included, except for the diffuse papillomatosis, where the sequence biliary papillomatosis to papillary carcinoma is proposed. We report a simultaneous case of gallbladder papillary neoplasia and intrahepatic duct carcinoma in situ associated with pancreaticobiliary maljunction (PBM). We proposed that double location, in our case, is more likely explained by a diffuse biliopancreatic tree disease leading to synchronous tumors arising in amenable duct. It was verified absence of continuity between gallbladder and intrahepatic bile duct site of involvement, absence of lymph node metastasis or venous involvement. This case report supports the concept of a proliferative and neoplastic process involving simultaneously the biliary tree and gallbladder associated with PBM.

## Introduction

Papillary carcinoma is composed predominantly of papillary structures lined by cuboidal or columnar epithelial cells often containing variable amounts of mucin [1-3]. It has been suggested [2-5] that papillary neoplasms in the biliary tract, including papillomatosis, are best considered the biliary counterpart to pancreatic IPMN (Intraductal Papillary Mucinous Neoplasia). Barton et al [1] suggest that papillary neoplasm, including papillomatosis, represent a non-invasive neoplasm that may give rise to invasive carcinoma. Zen et al [4] showed evidence that biliary papillomatosis and papillary cholangiocarcinoma with or without mucus hypersecretion potentially belong to a single tumor entity of biliary intraductal papillary neoplasm (BT-IPMN).

Pancreaticobiliary maljunction (PBM) is a congenital anomaly defined as a union of the pancreatic and biliary ducts outside of the duodenal wall which is often associated with hyper and dysplastic changes of the gallbladder [1, 4, 6]. The high risk of PBM for biliary tract cancer has been reported [4]. The carcinogenetic process in PBM has been explained by repeated damage of the biliary epithelium by the reflux of pancreatic and bile juice [7]. It is supposed that abnormalities of some oncogenes and cancer suppressor genes occur during each step of carcinogenesis [8].

Although double cancer of the gallbladder and extrahepatic bile duct associated with PBM has already been described [8], we have not find association with intrahepatic duct carcinoma. We report one case of double gallbladder papillary neoplasia with intrahepatic duct carcinoma in situ and PBM.

## Case Report

**Figure 1:**
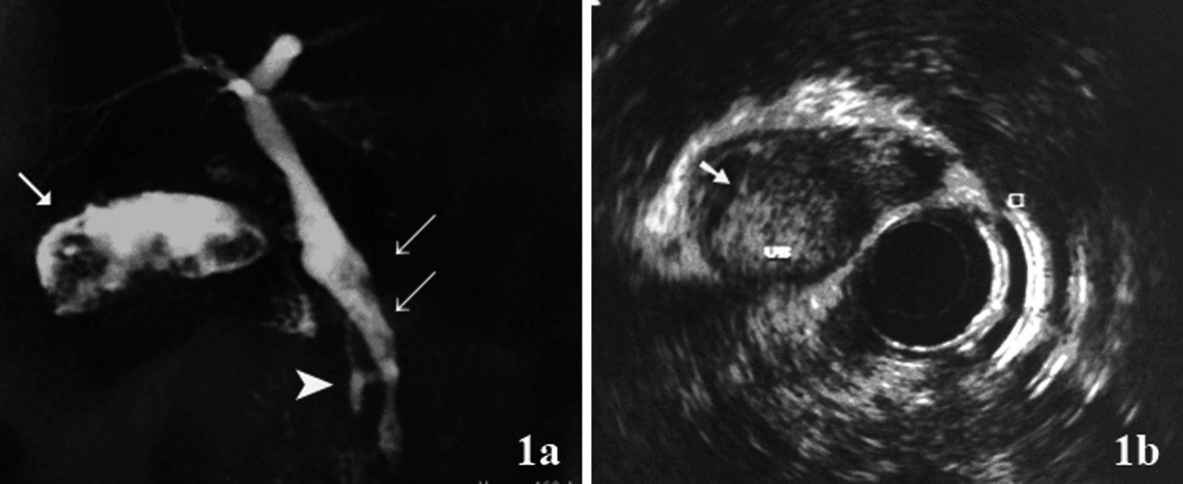
(a) Magnetic resonance cholangiopancreatography (MRCP) showed hyperdense structure within the gallbladder infundibulum, which were suggestive of tumor (arrow), bile duct dilatation and filling defect (double arrow) and pancreaticobiliary maljunction(arrow head); (b) Endoscopic ultrasound showed hyperechoic frond-like mass within the gallbladder (arrow).

**Figure 2:**
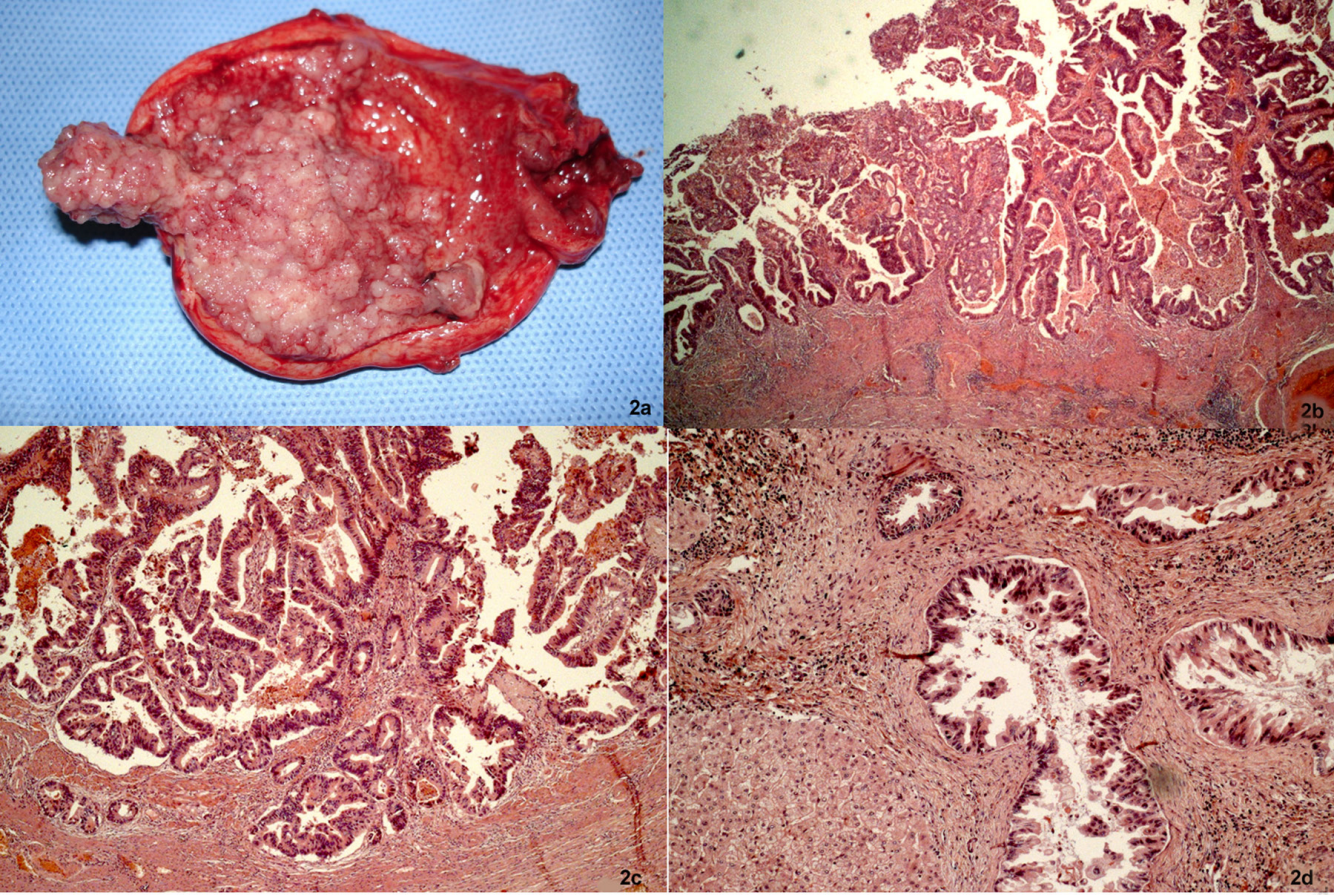
Cancer of the gallbladder and the bile duct. (a) Tumor within the gallbladder and the lumen was filled with mucin. (b, c) Papillary adenocarcinoma extending to the superficial portion of the muscle layer (H&E, × 25 and × 50). (d) The specimen of the liver showed carcinoma in situ in the segmental bile duct (H&E, × 100).

To describe the following case we have written permission of the patient. One female patient, 55-year-old, without biliary disease, was presented with transient icterus and pain and sensitivity on the epigastrium and right upper quadrant. Liver function tests were GGT = 92 IU/L, FA = 172 IU/L, TGO = 23 IU/L, TGP = 29 IU/L, BT = 0.5 mg/dL. In addition, tumor markers, including carbohydrate antigen 19-9 (CA 19.9), carcinoembryonic antigen (CEA) and Alfafetoprotein were within normal ranges. Magnetic resonance cholangiopancreatography (MRCP) showed bile duct dilatation and filling defect and pancreaticobiliary maljunction (Fig. 1a). Endoscopic ultrasound revealed dilated extrahepatic bile duct (CBD) with hyperechoic foci with no acoustic shadowing within, and a hyperechoic frond-like mass was noted within the infundibulum of gallbladder (Fig. 1b). Open cholecystectomy was performed and frozen section diagnosed gallbladder adenocarcinoma (Fig. 2a). The operation was completed with liver resection including segments 4b and 5, partial resection of the common bile duct and lymphadenectomy of the hepatic pedicle. Microscopic findings were gallbladder papillary carcinoma on the background of tubulopapillary adenoma (Fig. 2b, c) and carcinoma in situ in hepatic duct of segment 5 (Fig. 2d).

## Discussion

The carcinogenetic process in PBM has been explained by repeated damage and restoration of biliary epithelium by a mutual countercurrent of pancreatic and bile juice [6-8]. Regenerated epithelium gradually produces a variant accompanied by cellular atypical change, displaying a hyperplasia-dysplasia-carcinoma sequence [7]. Abnormalities of some oncogenes and cancer suppressor genes occur during each step of carcinogenesis. Molecular analysis shows multiple genetic mutations, among which K-ras gene activation and the p53 tumor suppressor gene inactivation in the mucosa of the gallbladder and bile duct are recognized as the most important keys for carcinogenesis in PBM [8].

We proposed that double location, in our case, is more likely explained by a diffuse biliopancreatic tree disease leading to synchronous tumors arising in amenable duct. It was verified absence of continuity between gallbladder and intrahepatic bile duct site of involvement, absence of lymph node metastasis or venous involvement. We remarked histopathological similarities, the coexistence of areas with varying degrees of epithelial dysplasia and carcinoma.

Similarities between biliary and pancreatic IPMN can be explained given the shared embryological development of the bile duct and the main pancreatic duct from the hepatic diverticulum in the foregut mesoderm, but important differences may exist [9]. For this reason, we suggest to consider papillary carcinoma of the gallbladder associated to intrahepatic carcinoma in situ as part of the same growth patterns of the invasive carcinoma for the others IPMN.

Various biliary neoplasms that have been described previously as adenomas, papillomatosis, adenocarcinomas, cholangiocarcinomas, cystic lesions and mucin-secreting lesions could be defined more clearly and consistently as BT-IPMN (Biliary Tract-IPMN) [1-3]. Albores-Saavedra et al [10] suggest that, in contrast to typical cholangiocarcinoma, invasive papillary cholangiocarcinoma grows towards the bile duct lumen prior to invading the bile duct wall. Mucin-hypersecreting papillomatosis of the gallbladder has been described [11-15]. The papillary carcinoma of the gallbladder as described in our case followed the same growth pattern toward the lumen prior to invading the gallbladder wall.

The most common presenting symptoms in patients with BT-IPMN were abdominal discomfort, obstructive jaundice, elevated serum liver enzymes level and epigastric pain [11-15]. We observed the same symptoms and large amounts of intraductal mucin were noted intraoperatively in our patient. This finding could explain the high levels of the canalicular enzymes and the dilatation of the common bile duct.

In conclusion, this case report supports the concept of a proliferative and neoplastic process involving simultaneously the biliary tree and gallbladder associated with PBM.
